# Deficient Glutathione in the Pathophysiology of Mycotoxin-Related Illness

**DOI:** 10.3390/toxins6020608

**Published:** 2014-02-10

**Authors:** Frederick T. Guilford, Janette Hope

**Affiliations:** 1 Your Energy Systems, LLC 555 Bryant St. #305, Palo Alto, CA 94301, USA; 2 Environmental Medicine, 304 W, Los Olivos Street, Santa Barbara, CA 93105, USA; E-Mail: janettehopemd@yahoo.com

**Keywords:** glutathione, mycotoxin, aflatoxin, ochratoxin, trichothecene, Nrf2 modification, genetic polymorphism, oxidative stress

## Abstract

Evidence for the role of oxidative stress in the pathophysiology of mycotoxin-related illness is increasing. The glutathione antioxidant and detoxification systems play a major role in the antioxidant function of cells. Exposure to mycotoxins in humans requires the production of glutathione on an “as needed” basis. Research suggests that mycotoxins can decrease the formation of glutathione due to decreased gene expression of the enzymes needed to form glutathione. Mycotoxin-related compromise of glutathione production can result in an excess of oxidative stress that leads to tissue damage and systemic illness. The review discusses the mechanisms by which mycotoxin-related deficiency of glutathione may lead to both acute and chronic illnesses.

## 1. Introduction

Mycotoxins are common contaminants of foods such as wheat, barley, grapes, coffee, spices, *etc*. and can affect the health of humans as well as livestock [1]. Aflatoxin, ochratoxins, trichothecenes, zearelenone, fumonisins, tremorgenic toxins, and ergot alkaloids are the mycotoxins with the largest agro-economic importance [2].

The fungal species *Fusarium* is one of the most common toxin-producing fungi of northern temperate regions and produces fumonisins, and trichothecenes. The trichothecenes are a large family of chemically related toxins and include T-2 toxinT-2, HT-2 toxin (HT-2), deoxynivalenol (DON), Diacetoxyscirpenol (DAS), Fusarenone-X (FUS-X), Nivalenol (NIV), diacetylnivalenol (DAS), neosolaniol and Zearalenone (ZEA [3]). Mycotoxins of the *Fusarium* species are generally of two types: (1) the nonestrogenic TCs such as DON, (NIV), T-2, and (DAS); (2) the mycoestrogens, including ZEA [3]. ZEA is a nonsteroidal, estrogenic mycotoxin and has been shown to be able to bind competitively to estrogen receptors [3]. The mycoestrogens can induce an estrogenic response in targeted cells [4], a finding which may enhance its synergistic effect with other with other trichothecenes (TCs) [3]. The black mold Stachybotrys chartarum is also known to produce trichothecenes such as Satratoxin-G (SG) [5].

Ochratoxin A (OTA), first discovered in 1965 in *Aspergillus ochraceus* has been subsequently reported in several *Aspergillus* and *Penicillium* species [6,7]. Aflatoxins, produced by the *Aspergillus* species are considered to be serious food contaminants worldwide [8], and aflatoxin B1 (AFB1) is a well-known carcinogen and genotoxic agent [9].

While there has been much research into the mechanisms of damage related to mycotoxins, the link between metabolism, toxicity and carcinogenicity of mycotoxins remains incompletely understood [10]. Mycotoxins occur in many foods and in low doses, mycotoxins such as aflatoxin generally do not cause health problems as they are bound to glutathione via interaction with the enzyme glutathione S-transferases (GST), which facilitates the excretion of mycotoxin [11]. Thus, both the availability of reduced glutathione and the GST enzymes are important in the prevention of mycotoxin-related pathology. Much of the work on mycotoxins has focused on their association with cancer. AFB1, a difurancoumarin mycotoxin produced by the mold *Aspergillus flavus*, is a potent, naturally occurring carcinogen [12]. The early observations that an epoxide of AFB1 created adducts with DNA led to the concept that mycotoxin-DNA adducts, such as AFB1-DNAs, initiate cancer—an observation which has been documented *in vitro* and *in vivo* [13,14].

In addition to carcinogenesis, toxicity is associated with mycotoxins. For example, in regard to aflatoxin, an AFB1 dialdehyde has been proposed as a toxin whose damage is also diminished by conjugation with GST [15,16]. Early research on aflatoxin led to the identification of DNA adducts as a significant factor in the genetic and general toxicity of this mycotoxin and many subsequent studies focused on mycotoxin-related adducts [17,18]. Some confusion regarding the mechanism of mycotoxin toxicity as the formation of adducts was not found with other mycotoxins, such as ochratoxin A (OTA) [19,20]. Interest in the mechanism of action of mycotoxins and especially OTA has increased with the availability of a Clinical Laboratory Improvement Amendments (CLIA) regulation-compliant registered laboratory test, which has identified OTA in the urine of humans with chronic illness [21,22]. One of the clinical studies identified OTA in 83% of over 100 individuals tested with chronic illness and a history of water-damaged building exposure [22]. These findings suggest that an increased understanding of the pathophysiology of mycotoxin-related illness may be useful in designing treatment for these problems.

Continued studies on OTA led to the observation that oxidative stress was a possible key factor in the toxicity and genotoxicity of OTA [23,24,25,26]. OTA was shown to increase the formation of oxidative products of lipids, with increased production of malondialdehyde (MDA), a product of the interaction of polyunsaturated lipids and free radicals [24,27,28]. It was shown that the toxicity of OTA could be decreased by maintaining glutathione production with *N*-acetyl cysteine (NAC), which decreased reactive oxygen species (ROS) and 8-oxoguanine formation. These findings suggested that cellular glutathione (GSH) levels play a significant role in limiting the toxicity of the mycotoxin OTA [29].

Recent reviews suggest both direct, DNA adduct-mediated mechanism [30] and an oxidative stress-mediated mechanism as causes of the carcinogenicity of OTA. It appears that that oxidative stress plays a significant role in the toxicity of mycotoxins [31,32,33,34]. The focus of this review is the effect of mycotoxins on intracellular glutathione and the multifactorial function of glutathione as an antioxidant and detoxifier as well as the loss of glutathione as a significant factor in the pathogenesis of illnesses related to mycotoxin accumulation.

## 2. Oxidative Stress

The concept of oxidative stress has developed from the free radical theory of oxygen toxicity developed in the 1950s with studies such as those by Rebecca Gerschman showing that oxygen poisoning and X-irradiation can both produce oxygen-derived free radicals [35,36]. It has been known since the writing by Lavoisier in the late 1700s that oxygen can cause damage to the lipids of animal cells [37], as recounted in [38]. The studies of ionizing radiation demonstrated that free radicals of oxygen can be generated not only by ionizing radiation, but were also formed during normal oxidative metabolism. These processes formed radicals such as hydroxyl radicals OH^•^, hydroperoxyl radicals H O_2_^•^ and peroxide H_2_O_2_ [35].

Oxidative stress occurs when there is an increase in the production of free radicals and reactive metabolites, which are termed oxidants and which exceed the ability of the cell to eliminate the oxidants by protective mechanisms, termed antioxidants. This excess of oxidants leads to the damage to the biomolecules of cells, tissues and organs with a potentially harmful impact on the whole organism. The constant interaction between oxidants and antioxidant function is part of the normal function of cells and can lead to differences in glutathione concentrations within cellular and tissue compartments [39].

## 3. Glutathione Sources in the Body

Composed of glutamine, cysteine, and glycine and utilized as an antioxidant, GSH is the main nonprotein thiol responsible for cellular homeostasis and maintenance of the cellular redox balance [40,41]. Existing in two forms, oxidized (GSSG) and reduced/free form (GSH), only GSH exhibits antioxidant activity. During oxidative stress, GSH is utilized to neutralize reactive oxygen species leading to the formation of GSSG. GSSG is the by-product of the free radical-scavenging activity of GSH and lacks antioxidant function [40,41].

**Figure 1:**
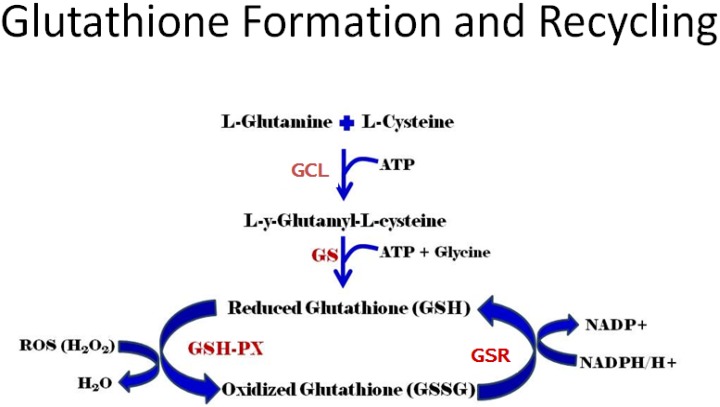
Production of GSH occurs by two mechanisms, *de novo* synthesis and recycling of GSSG. *De novo* synthesis occurs in a two-step reaction catalyzed by two separate enzymes, glutamine-cysteine ligase (GCL) and glutathione synthase (GS). The enzyme glutathione reductase (GSR) catalyzes the reduction of GSSG back to GSH. Glutathione peroxidase (GSH-PX) is a selenium-based enzyme that reduces hydrogen peroxide (H_2_O_2_) to water.

Production of GSH occurs by two mechanisms, *de novo* synthesis and recycling of GSSG. *De novo* synthesis occurs in a two-step reaction catalyzed by two separate enzymes, glutamine-cysteine ligase (GCL) and glutathione synthase (GS), as shown in Figure 1. The first step in the reaction is catalyzed by GCL, a heterodimer made up of a catalytic subunit (GCLC) that possesses the enzyme’s active site and performs the actual amino acid linkage, and a modulating subunit (GCLM) that regulates the activity of GCLC, as reviewed in Figure 1. This first step is rate limiting, with cysteine availability being the rate-limiting component [40,41]. In the final step of *de novo* GSH synthesis, glycine is linked to the dimer formed in the previous step reaction by GS. *De novo* synthesis of GSH is regulated by negative feedback.

Gamma-glutamyltransferase 1 (GGT1) is a membrane-bound enzyme that catalyzes the breakdown of extracellular GSH into glutamate and cysteinyl-glycine, providing raw materials for the *de novo* of GSH [42,43,44].

Finally, GSH can be obtained by the reduction of GSSG via the glutathione reductase (GSR) enzyme. This reaction requires NADPH and forms two GSH molecules from one GSSG molecule [40,41]. It is now known that a number of interactions may occur that prevent the *de novo* formation of GSH. For example, it has been shown that glutathione is decreased due to an inability to produce glutathione in the extracellular lung fluid of children with chronic asthma [45] and in the macrophages of adults with human immunodeficiency virus (HIV) [46].

### 3.1. Gene Control of GSH Production

Glutathione biosynthesis, glutathione peroxidases, glutathione S-transferases and glutathione S-conjugate efflux pumps function in a coordinated fashion to facilitate a coordinated response to oxidative stress. Regulation of this response is facilitated by the antioxidant responsive element (ARE) which is located in the promoters of many of the genes that are induced by oxidative and chemical stress [47].

**Figure 2:**
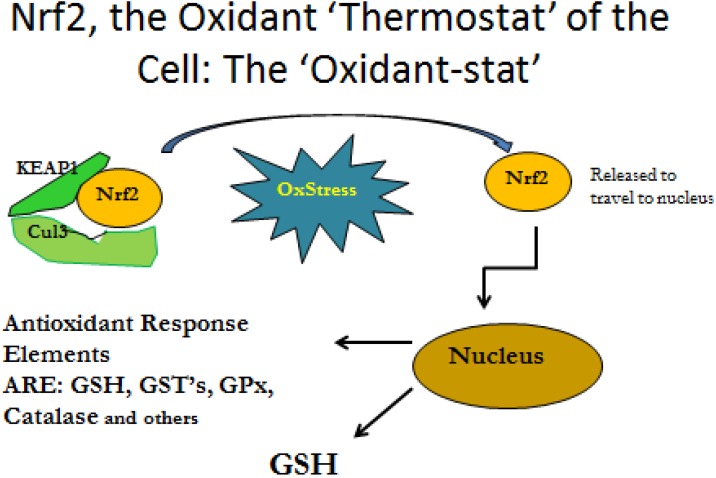
The transcription factor nuclear factor (erythroid-derived 2)-like 2 (Nrf2) plays a key role in redox regulation. Nrf2 is expressed by the majority of cell types; it is anchored in the cytosol by an inhibitory protein, Kelch-like ECH-associated protein (Keap1). During oxidative stress, Nrf2 is released and transfers to the nucleus, where it binds and activates the antioxidant response elements (ARE) and upregulates the expression of several genes associated with antioxidant defense and glutathione (GSH) synthesis.

The investigation of the mechanism of glutathione deficiency in the lungs children with asthma has shown that dysregulation of glutathione-related antioxidant defense occurs due to dysfunction of a transcription factor that stimulates gene expression of the enzymes related to the antioxidant defense and glutathione production in cells. The transcription factor nuclear factor (erythroid-derived 2)-like 2 (Nrf2) is a transcription factor with a basic leucine zipper motif that plays a key role in redox regulation. Nrf2 is expressed the majority of cell types, where it is anchored in the cytosol by an inhibitory protein, Kelch-like ECH-associated protein (Keap1). With an increase in oxidative stress, Nrf2 is released and translocates to the nucleus, where it binds and activates the ARE and upregulates several genes associated with glutathione synthesis and antioxidant defense (Figure 2) [45,48].

It has been shown that compromise of Nrf2 function can occur due to modification of Nrf2 after production, even when its production is upregulated [45]. It appears that excess oxidative stress causes posttranslational modification of Nrf2. Without Nrf2 function, the genes that form GCL and GS are not expressed with the result that GSH is not formed. The damaging effects of Nrf2 modification in the lung, as an example, may increase the susceptibility of the affected tissues to the oxidative stress of the oxygen environment, as well as compromise the function of epithelial cells and phagocytes, such as granulocytic leukocytes and monocytes/macrophages [49,50].

The importance of Nrf2 function is shown in an animal model in which the upregulation of Nrf2 will protect against dietary carcinogen diethylnitrosamine (DENA)-induced liver carcinogenesis [51].

Similar compromise of gene up-expression has been shown to occur in macrophages obtained from individuals with HIV infection [52]. This study demonstrates that without function of the genes needed to produce glutathione supplying the building block of glutathione production, NAC is much less efficient than supplying the whole molecule of glutathione using a liposomal encapsulation of glutathione [46].

### 3.2. Cytokine Influence on GSH Production

In the macrophages of individuals with HIV, the gene expression for the formation of the enzymes of GSH synthesis GCLC, GCLM, GSS, and GGT1 appear to be down-regulated [53]. Down-regulation of the GCLC gene results in decreased synthesis of GSH that is related to an increased formation of an inflammatory cytokine called transforming growth factor-β (TGF-β) [54,55]. It has been shown that decreased levels of GSH will stimulate production of TGF F-β and that when GSH is replenished, the production of TGF-β will be decreased [56].

### 3.3. Glutathione S-Transferase

Nrf2 transcription also controls the formation of Glutathione S-transferases (GSTs). Glutathione S-transferase is a multi-isoform family of enzymes that detoxify endobiotic and xenobiotic compounds. Each subunit of the dimeric GST enzyme has an active site composed of two distinct functional regions: a hydrophilic G-site, which binds the physiological substrate glutathione, and an adjacent H-site which provides a hydrophobic environment for the binding of structurally diverse electrophilic substrates [57]. This structure allows GSTs to catalyze the nucleophilic attack of glutathione (GSH) [58] on electrophilic substrates, which decreases the electrophile’s reactivity with cellular macromolecules [57]. In humans and rodents, the soluble GSTs are collectively expressed in relatively large amounts, and may comprise as much as 4% of total soluble protein in the liver [58].

### 3.4. GST Genetic Polymorphisms

The level and molecular form of glutathione is a key determinant of the cellular response to oxidative stress [59] and the interaction with glutathione S-transferase (GST) will affect the level of glutathione [47]. GSTs function by conjugating reduced GSH and catalyzing the attack on foreign compounds or oxidative stress products, generally forming less-reactive materials that can be readily excreted. There are three distinct families of GSTs in mammals: cytosolic, mitochondrial and membrane-associated proteins. Cytosolic GSTs represent the largest family and is subdivided into seven classes based on their amino acid sequence (mu, pi, theta, alpha, sigma, omega and zeta). The GST within a particular class typically share more than 40% of their identity, but at least five of the human GST genes display functional polymorphisms. These polymorphisms are likely to contribute to the difference between individuals in responses to xenobiotics and clearance of oxidative stress products and have been associated with various pathologies such as cancer, and cardiovascular or respiratory diseases [60]. Individuals homozygous for the GSTM1 deletion polymorphism represent approximately 50% of the population and lack expression of this enzyme in any tissue in the body [58]. The implication of GST polymorphisms may be significant as studies suggest that that loss of mu, pi and theta GST genes increase susceptibility to inflammatory diseases, and a genetic deficiency of GSTM1 is associated with increased susceptibility to ozone-related asthma [61] and to susceptibility to AFB1-mediated damage to human liver cells [62]. These authors suggested that individuals with GSTM1 polymorphism may benefit from antioxidant supplementation. The combination of GSTM1 null and GSTP1 Val was significantly associated with an increased risk of lung cancer [63] and hepatocellular carcinoma [64]. An inverse relationship between plasma selenium level, an indicator of the function of glutathione peroxidase and AFB1–albumin adducts were observed to be statistically significant among individuals with null genotypes of GSTM1 and GSTT1, but not among the non-null genotypes [65]. In carriers of hepatitis B virus, there was an increased risk of HCC in null genotypes of GSTM1 and T1 but not among individuals who had non-null genotypes [66]. Additionally, GSTM1 null genotype is a risk factor for Alzheimer’s disease [67] and T-2 can suppress drug-metabolizing enzymes such as the GSTs [68] and related oxidative stress-associated pathways [68].

## 4. Identification of Mycotoxin-Related Oxidant Stress

OTA causes a variety of toxic presentations in animals with renal toxicity—a prominent and consistent finding [69,70]—with these cells showing inhibition of protein synthesis [71], impairment of energetic metabolism and changes in cell phenotype in renal cells [72], as well as increased oxidative stress [24,26,72].

These findings led to the use of toxicogenomic profiles for analysis of the cellular responses to OTA. The evidence that OTA caused increased oxidative stress led to the expectation that gene expression profiles after exposure to OTA in animals would show the induction of many genes responsive to exposure to oxidative stress. Surprisingly, in tissue samples expected to be damaged by OTA, such as kidney, it was found that OTA caused a down-regulation of genes expected to be stimulated by oxidant exposure. A study of rats exposed chronically to OTA, even at doses that resulted in 25% of the animals developing kidney tumor formation, the gene expression profile showed down-regulation of the antioxidant genes [73]. In these studies, OTA induced tissue-specific responses identified in kidney *versus* liver. Applying toxigenomic assessment to the studies of OTA revealed several data to help explain the mechanism of action of OTA. For example, OTA induced strong down-regulation of regucalcin, which interferes with intracellular calcium regulation, a finding associated with nephrotoxic and nephrocarcinogenic compounds such as cisplatin [74,75]. It was also shown that renal transport proteins that facilitate the urinary excretion of OTA, as well as multidrug resistance (MDR) proteins, were down-regulated [73]. The tissue injury associated with OTA was accompanied by a disruption of pathways related to the transcription factors hepatocyte nuclear factor 4 alpha (HNF4α) and nuclear factor-erythroid 2-related factor 2 (Nrf2) [73]. Many Nrf2-regulated genes are involved in chemical detoxification and antioxidant defense [76]. The findings suggested that depletion of these genes results in a decrease of the oxidant defense mechanism that was a likely cause of damage to both cell and genetic functions in cells [73].

It appears that an active transport mechanism concentrates OTA in the kidney, which results in the toxicity of OTA [77,78]. While the impact of OTA appears to be related to its concentration in the kidney, sufficiently high levels of OTA will cause oxidant damage in any organ tissue [79].

Exposure to OTA can down-regulate the formation of GCLC, the rate-limiting enzyme in GSH synthesis, which will result in a reduction in the intracellular level of GSH [73]. The block of the GCLC enzyme may explain the decrease of GSH previously found in OTA-treated cell cultures [29]. In addition to decreasing GSH, OTA inhibition of Nrf2 expression will decrease the production of the GST isoform GSTP1, which facilitates the detoxification of the lipid peroxidation product 4-hydoxynonenal (4-HNE) by facilitating the conjugation of 4-HNE with GSH. The reactive chemical 4-HNE is known to bind and form adducts to macromolecules including DNA [80], and cell culture studies demonstrate that Nrf2 and glutathione are needed to protect cells from 4-HNE and lipid peroxidation toxicity [81]. Thus, the formation of GST and GCL are central to the detoxification of OTA and the loss of formation of the antioxidant, detoxification enzymes related to glutathione suggests that OTA inhibits its own detoxification [82].

The findings of increased oxidant stress related to decreased expression of Nrf2 in kidney cells suggests that direct binding of the mycotoxin to DNA is not needed to create DNA damage [79]. The down-regulation of Nrf2 genes results in a decrease in antioxidant defense is due to the decreased expression of Nrf2-related antioxidant genes and thus, the oxidative stress induced by OTA may account for both the genetic damage and the cytotoxicy related to OTA [79]. Thus, the oxidative damage mediated by OTA may be divided into the direct (covalent DNA adduction) and indirect (oxidative DNA damage) mechanisms [83]. Trichothecene type mycotoxins such as T-2 toxin have also been shown to cause cell damage by increasing oxidative stress and depleting glutathione [84].

## 5. Mycotoxin Effect on Immunity is Oxidative Stress Related

AFB1 mainly affects cell-mediated immunity and has also been shown to increase inflammation [85]. T-2 toxin, can decrease the function of the innate immune system [86]. Both AFB1 and FB1 are carcinogenic and modulate immunity [18]. Both AFB1 and FB1 increase ROS and stimulate biomolecular oxidative damage in spleen mononuclear cells; the combination appears to have even stronger pro-oxidant activity [87]. A similar immunosuppression has been demonstrated by the combination of DON, and FB1 [88,89] combination increase ROS in spleen mononuclear cells [87]. These results demonstrate that immune cells are affected by mycotoxins and that oxidative stress is a major component of the damage in these cells.

Additional suggestion that the interaction between glutathione and mycotoxin plays an important role in immune cell function comes from studies of the mycotoxins gliotoxin and patulin which show that in murine dendritic cells (antigen-presenting macrophage cells), the mycotoxins depleted glutathione in a concentration-dependent fashion [90]. Respiratory exposure to gliotoxin or ingestion of patulin increased the asthma-like phenotype in an animal model and additionally increased chronic airway inflammation [90]. Both gliotoxin and patulin have been shown to shift the Th1/Th2 balance toward a Th2 response [91,92]. Mycotoxins were shown to decrease glutathione in PBMC and alveolar cells [92,93].

The central role of glutathione in a variety of cell functions related to immune defense has been demonstrated in previous studies. It has been shown that a decrease in glutathione in antigen-presenting cells correlates with increased Th2 response [94,95]. Decreased glutathione found in macrophage cells from children with chronic asthma has been shown to be related to decreased bacterial phagocytosis [96]. In the children with chronic asthma, the decrease in glutathione was shown to be related to post-translational modification of Nrf2, which resulted in decreased gene expression of the enzymes of glutathione production. Decreased glutathione and decreased macrophage defense against intracellular infection with *Mycobacterium tuberculosis* occurs in the macrophages of individuals with HIV [52]. The decrease in glutathione in the macrophage of HIV^+^ individuals was shown to be due to a decrease in the gene expression of the enzymes of glutathione production [52].

Restoration of glutathione levels in the mycotoxin exposed mouse dendritic cells using NAC or glutathione ethyl ester restored IL-12 secretion and prevented the mycotoxin-induced increase of airway inflammation and airway hyperreactivity [90]. Similarly, restoration of glutathione using NAC in the macrophages of children with asthma restored phagocytosis in the *ex vivo* model [96]. The loss of glutathione in the HIV^+^ macrophages has been shown to be related to decreased cell defense against *Mycobacterium tuberculosis* replication [52].

## 6. Conclusions

Evidence that oxidative stress is a significant factor in the pathophysiology of mycotoxin-related illness is accumulating. The evidence that OTA-related oxidative stress is a factor in nephrocarcinogenicity has been demonstrated in a comprehensive review [31]. The demonstration that the oxidative stress associated with mycotoxins can be related to direct suppression of GCLC gene function, post-translational modification of Nrf2 or the presence of excess TGF-β suggests that the lack of glutathione may be due to the inability of mycotoxin-affected cells to adequately form glutathione. Decreased function of the enzymes of glutathione production results in a microenvironment depleted of glutathione on a chronic basis. In humans, deficiency of glutathione can lead to chronic conditions [97], including chronic asthma [45]. A decrease in glutathione due to mycotoxin-related depletion may contribute to the range of conditions associated with mycotoxin accumulation [22]. The lack of function of the enzymes of glutathione production in these conditions suggests that more efficient resolution of the effects of glutathione depletion may require the administration of the complete molecule as demonstrated *in vitro* in the HIV^+^ macrophage study [52]. These observations echo an early report regarding the benefit of glutathione in the management of aflatoxin-related hepatocellular carcinoma in an animal model, which proposed that administration of the intact glutathione molecule was needed for benefit of the use of glutathione [98]. Anecdotal reports suggest that studies using liposomal glutathione in the management of mycotoxin-related conditions may be warranted [21,99].

## References

[1] Council for Agricultural Science and Technology (2002). Mycotoxins: Risks in Plant, Animal, and Human Systems.

[2] Hussein H.S., Brasel J.M. (2001). Toxicity, metabolism, and impact of mycotoxins on humans and animals. Toxicology.

[3] Yazar S., Omurtag G.Z. (2008). Fumonisins, trichothecenes and zearalenone in cereals. Int. J. Mol. Sci..

[4] Sondergaard T.E., Klitgaard L.G., Purup S., Kobayashi H., Giese H., Sorensen J.L. (2012). Estrogenic effects of fusarielins in human breast cancer cell lines. Toxicol. Lett..

[5] Carey S.A., Plopper C.G., Hyde D.M., Islam Z., Pestka J.J., Harkema J.R. (2012). Satratoxin-G from the black mold *Stachybotrys chartarum* induces rhinitis and apoptosis of olfactory sensory neurons in the nasal airways of rhesus monkeys. Toxicol. Pathol..

[6] Van der Merwe K.J., Steyn P.S., Fourie L., Scott D.B., Theron J.J. (1965). Ochratoxin A, a toxic metabolite produced by *Aspergillus ochraceus* Wilh. Nature.

[7] Larsen T.O., Svendsen A., Smedsgaard J. (2001). Biochemical characterization of ochratoxin A—Producing strains of the genus *penicillium*. Appl. Environ. Microbiol..

[8] Abbas H.K. (2005). Aflatoxin and Food Safety.

[9] Wang J.S., Groopman J.D. (1999). DNA damage by mycotoxins. Mutat. Res..

[10] Ellis E.M. (2009). Protection against aflatoxin B1 in rat—A new look at the link between toxicity, carcinogenicity, and metabolism. Toxicol. Sci..

[11] Roebuck B.D., Johnson D.N., Sutter C.H., Egner P.A., Scholl P.F., Friesen M.D., Baumgartner K.J., Ware N.M., Bodreddigari S., Groopman J.D. (2009). Transgenic expression of aflatoxin aldehyde reductase (akr7a1) modulates aflatoxin B1 metabolism but not hepatic carcinogenesis in the rat. Toxicol. Sci..

[12] Eaton D.L., Gallagher E.P. (1994). Mechanisms of aflatoxin carcinogenesis. Annu. Rev. Pharmacol. Toxicol..

[13] Croy R.G., Essigmann J.M., Reinhold V.N., Wogan G.N. (1978). Identification of the principal aflatoxin B1-DNA adduct formed *in vivo* in rat liver. Proc. Natl. Acad. Sci. USA.

[14] Essigmann J.M., Croy R.G., Nadzan A.M., Busby W.F., Reinhold V.N., Büchi G., Wogan G.N. (1977). Structural identification of the major DNA adduct formed by aflatoxin B1 *in vitro*. Proc. Natl. Acad. Sci. USA.

[15] Hayes J.D., Pulford D.J., Ellis E.M., McLeod R., James R.F., Seidegard J., Mosialou E., Jernstrom B., Neal G.E. (1998). Regulation of rat glutathione S-transferase A5 by cancer chemopreventive agents: Mechanisms of inducible resistance to aflatoxin B1. Chem. Biol. Interact..

[16] Ilic Z., Crawford D., Vakharia D., Egner P.A., Sell S. (2010). Glutathione-S-transferase A3 knockout mice are sensitive to acute cytotoxic and genotoxic effects of aflatoxin B1. Toxicol. Appl. Pharmacol..

[17] Dvorackova I. (1989). Aflatoxins & Human Health.

[18] Wild C.P., Gong Y.Y. (2010). Mycotoxins and human disease: A largely ignored global health issue. Carcinogenesis.

[19] Mally A., Zepnik H., Wanek P., Eder E., Dingley K., Ihmels H., Volkel W., Dekant W. (2004). Ochratoxin A: Lack of formation of covalent DNA adducts. Chem. Res. Toxicol..

[20] Turesky R.J. (2005). Perspective: Ochratoxin A is not a genotoxic carcinogen. Chem. Res. Toxicol..

[21] Hope J.H., Hope B.E. (2012). A review of the diagnosis and treatment of ochratoxin a inhalational exposure associated with human illness and kidney disease including focal segmental glomerulosclerosis. J. Environ. Public Health.

[22] Brewer J.H., Thrasher J.D., Straus D.C., Madison R.A., Hooper D. (2013). Detection of mycotoxins in patients with chronic fatigue syndrome. Toxins.

[23] Gautier J.C., Holzhaeuser D., Markovic J., Gremaud E., Schilter B., Turesky R.J. (2001). Oxidative damage and stress response from ochratoxin a exposure in rats. Free Radic. Biol. Med..

[24] Petrik J., Zanic-Grubisic T., Barisic K., Pepeljnjak S., Radic B., Ferencic Z., Cepelak I. (2003). Apoptosis and oxidative stress induced by ochratoxin a in rat kidney. Arch. Toxicol..

[25] Schilter B., Marin-Kuan M., Delatour T., Nestler S., Mantle P., Cavin C. (2005). Ochratoxin A: Potential epigenetic mechanisms of toxicity and carcinogenicity. Food Addit. Contam..

[26] Kamp H.G., Eisenbrand G., Janzowski C., Kiossev J., Latendresse J.R., Schlatter J., Turesky R.J. (2005). Ochratoxin A induces oxidative DNA damage in liver and kidney after oral dosing to rats. Mol. Nutr. Food Res..

[27] Omar R.F., Hasinoff B.B., Mejilla F., Rahimtula A.D. (1990). Mechanism of ochratoxin A stimulated lipid peroxidation. Biochem. Pharmacol..

[28] Sauvant C., Holzinger H., Gekle M. (2005). The nephrotoxin ochratoxin A induces key parameters of chronic interstitial nephropathy in renal proximal tubular cells. Cell. Physiol. Biochem..

[29] Schaaf G.J., Nijmeijer S.M., Maas R.F., Roestenberg P., de Groene E.M., Fink-Gremmels J. (2002). The role of oxidative stress in the ochratoxin A—Mediated toxicity in proximal tubular cells. Biochim. Biophys. Acta.

[30] Pfohl-Leszkowicz A., Manderville R.A. (2012). An update on direct genotoxicity as a molecular mechanism of ochratoxin a carcinogenicity. Chem. Res. Toxicol..

[31] Marin-Kuan M., Ehrlich V., Delatour T., Cavin C., Schilter B. (2011). Evidence for a role of oxidative stress in the carcinogenicity of ochratoxin A. J. Toxicol..

[32] Alpsoy L., Yildirim A., Agar G. (2009). The antioxidant effects of vitamin A, C, and E on aflatoxin B1-induced oxidative stress in human lymphocytes. Toxicol. Ind. Health.

[33] Zhang L., Ye Y., An Y., Tian Y., Wang Y., Tang H. (2010). Systems responses of rats to aflatoxin B1 exposure revealed with metabonomic changes in multiple biological matrices. J. Proteome Res..

[34] Akcam M., Artan R., Yilmaz A., Ozdem S., Gelen T., Naziroglu M. (2013). Caffeic acid phenethyl ester modulates aflatoxin B1-induced hepatotoxicity in rats. Cell Biochem. Funct..

[35] Gerschman R., Gilbert D., Nye S.W., Dwyer P., Fenn W.O. (1954). Oxygen poisoning and x-irradiation: A mechanism in common. Science.

[36] Commoner B., Townsend J., Pake G.E. (1954). Free radicals in biological materials. Nature.

[37] Lavoisier A.L. (1789). Traité élémentaire de chimie: Présenté dans un ordre nouveau et d’après les découvertes modernes.

[38] Weissmann G. (2010). Free radicals can kill you: Lavoisier’s oxygen revolution. FASEB J..

[39] Fitzpatrick A.M., Jones D.P., Brown L.A. (2012). Glutathione redox control of asthma: From molecular mechanisms to therapeutic opportunities. Antioxid. Redox Signal..

[40] Wu G., Fang Y.Z., Yang S., Lupton J.R., Turner N.D. (2004). Glutathione metabolism and its implications for health. J. Nutr..

[41] Forman H.J., Zhang H., Rinna A. (2009). Glutathione: Overview of its protective roles, measurement, and biosynthesis. Mol. Asp. Med..

[42] Zhang H., Forman H.J., Choi J. (2005). Gamma-glutamyl transpeptidase in glutathione biosynthesis. Methods Enzymol..

[43] Hanigan M.H. (1998). Gamma-glutamyl transpeptidase, a glutathionase: Its expression and function in carcinogenesis. Chem. Biol. Interact..

[44] Heisterkamp N., Groffen J., Warburton D., Sneddon T.P. (2008). The human gamma-glutamyltransferase gene family. Hum. Genet..

[45] Fitzpatrick A.M., Stephenson S.T., Hadley G.R., Burwell L., Penugonda M., Simon D.M., Hansen J., Jones D.P., Brown L.A. (2011). Thiol redox disturbances in children with severe asthma are associated with posttranslational modification of the transcription factor nuclear factor (erythroid-derived 2)-like 2. J. Allergy Clin. Immunol..

[46] Morris D., Guerra C., Khurasany M., Guilford F., Saviola B., Huang Y., Venketaraman V. (2013). Glutathione supplementation improves macrophage functions in HIV. J. Interferon Cytokine Res..

[47] Hayes J.D., McLellan L.I. (1999). Glutathione and glutathione-dependent enzymes represent a co-ordinately regulated defence against oxidative stress. Free Radic. Res..

[48] Reddy N.M., Kleeberger S.R., Yamamoto M., Kensler T.W., Scollick C., Biswal S., Reddy S.P. (2007). Genetic dissection of the Nrf2-dependent redox signaling-regulated transcriptional programs of cell proliferation and cytoprotection. Physiol. Genomics.

[49] Comhair S.A., Erzurum S.C. (2010). Redox control of asthma: Molecular mechanisms and therapeutic opportunities. Antioxid. Redox Signal..

[50] Fitzpatrick A.M., Holguin F., Teague W.G., Brown L.A. (2008). Alveolar macrophage phagocytosis is impaired in children with poorly controlled asthma. J. Allergy Clin. Immunol..

[51] Bishayee A., Bhatia D., Thoppil R.J., Darvesh A.S., Nevo E., Lansky E.P. (2011). Pomegranate-mediated chemoprevention of experimental hepatocarcinogenesis involves Nrf2-regulated antioxidant mechanisms. Carcinogenesis.

[52] Morris D., Guerra C., Donohue C., Oh H., Khurasany M., Venketaraman V. (2012). Unveiling the mechanisms for decreased glutathione in individuals with HIV infection. Clin. Dev. Immunol..

[53] Bakin A.V., Stourman N.V., Sekhar K.R., Rinehart C., Yan X., Meredith M.J., Arteaga C.L., Freeman M.L. (2005). Smad3-ATF3 signaling mediates TGF-beta suppression of genes encoding Phase II detoxifying proteins. Free Radic. Biol. Med..

[54] Franklin C.C., Rosenfeld-Franklin M.E., White C., Kavanagh T.J., Fausto N. (2003). TGFβ1-induced suppression of glutathione antioxidant defenses in hepatocytes: Caspase-dependent post-translational and caspase-independent transcriptional regulatory mechanisms. FASEB J..

[55] Liu R.M., Gaston Pravia K.A. (2010). Oxidative stress and glutathione in TGF-beta-mediated fibrogenesis. Free Radic. Biol. Med..

[56] Armstrong R.N. (1997). Structure, catalytic mechanism, and evolution of the glutathione transferases. Chem. Res. Toxicol..

[57] Eaton D.L., Bammler T.K. (1999). Concise review of the glutathione S-transferases and their significance to toxicology. Toxicol. Sci..

[58] Yin Z., Ivanov V.N., Habelhah H., Tew K., Ronai Z. (2000). Glutathione S-transferase p elicits protection against H_2_O_2_-induced cell death via coordinated regulation of stress kinases. Cancer Res..

[59] Hayes J.D., Flanagan J.U., Jowsey I.R. (2005). Glutathione transferases. Annu. Rev. Pharmacol. Toxicol..

[60] Romieu I., Sienra-Monge J.J., Ramirez-Aguilar M., Moreno-Macias H., Reyes-Ruiz N.I., Estela del Rio-Navarro B., Hernandez-Avila M., London S.J. (2004). Genetic polymorphism of GSTM1 and antioxidant supplementation influence lung function in relation to ozone exposure in asthmatic children in Mexico City. Thorax.

[61] Gross-Steinmeyer K., Stapleton P.L., Tracy J.H., Bammler T.K., Strom S.C., Eaton D.L. (2010). Sulforaphane- and phenethyl isothiocyanate-induced inhibition of aflatoxin B1-mediated genotoxicity in human hepatocytes: Role of GSTM1 genotype and CYP3A4 gene expression. Toxicol. Sci..

[62] Wang J., Deng Y., Cheng J., Ding J., Tokudome S. (2003). GST genetic polymorphisms and lung adenocarcinoma susceptibility in a Chinese population. Cancer Lett..

[63] Deng Z.L., Wei Y.P., Ma Y. (2005). Polymorphism of glutathione S-transferase *mu* 1 and *theta* 1 genes and hepatocellular carcinoma in southern Guangxi, China. World J. Gastroenterol..

[64] Chen S.Y., Chen C.J., Tsai W.Y., Ahsan H., Liu T.Y., Lin J.T., Santella R.M. (2000). Associations of plasma aflatoxin B1-albumin adduct level with plasma selenium level and genetic polymorphisms of glutathione S-transferase M1 and T1. Nutr. Cancer.

[65] Sun C.A., Wang L.Y., Chen C.J., Lu S.N., You S.L., Wang L.W., Wang Q., Wu D.M., Santella R.M. (2001). Genetic polymorphisms of glutathione S-transferases M1 and T1 associated with susceptibility to aflatoxin-related hepatocarcinogenesis among chronic hepatitis B carriers: A nested case-control study in Taiwan. Carcinogenesis.

[66] Piacentini S., Polimanti R., Squitti R., Ventriglia M., Cassetta E., Vernieri F., Rossini P.M., Manfellotto D., Fuciarelli M. (2012). GSTM1 null genotype as risk factor for late-onset Alzheimer’s disease in italian patients. J. Neurol. Sci..

[67] Doi K., Uetsuka K. (2011). Mechanisms of mycotoxin-induced neurotoxicity through oxidative stress-associated pathways. Int .J. Mol. Sci..

[68] Boorman G.A. (1989). National Toxicology Program. Toxicology and carcinogenesis studies of ochratoxin A (CAS No. 303-47-9) in F344/N rats (gavage studies). Natl. Toxicol. Program Tech. Rep. Ser..

[69] Dietrich D.R., Heussner A.H., O’Brien E. (2005). Ochratoxin A: Comparative pharmacokinetics and toxicological implications (experimental and domestic animals and humans). Food Addit. Contam..

[70] Dirheimer G., Creppy E.E. (1991). Mechanism of action of ochratoxin A. IARC Sci. Publ..

[71] Gekle M., Sauvant C., Schwerdt G. (2005). Ochratoxin A at nanomolar concentrations: A signal modulator in renal cells. Mol. Nutr. Food Res..

[72] Marin-Kuan M., Nestler S., Verguet C., Bezencon C., Piguet D., Mansourian R., Holzwarth J., Grigorov M., Delatour T., Mantle P. (2006). A toxicogenomics approach to identify new plausible epigenetic mechanisms of ochratoxin A carcinogenicity in rat. Toxicol. Sci..

[73] Huang Q., Dunn R.T., Jayadev S., DiSorbo O., Pack F.D., Farr S.B., Stoll R.E., Blanchard K.T. (2001). Assessment of cisplatin-induced nephrotoxicity by microarray technology. Toxicol. Sci..

[74] Misawa H., Yamaguchi M. (2001). Involvement of nuclear factor-1 (NF1) binding motif in the regucalcin gene expression of rat kidney cortex: The expression is suppressed by cisplatin administration. Mol. Cell. Biochem..

[75] Lee J.M., Johnson J.A. (2004). An important role of Nrf2—Are pathway in the cellular defense mechanism. J. Biochem. Mol. Biol..

[76] Schwerdt G., Bauer K., Gekle M., Silbernagl S. (1996). Accumulation of ochratoxin A in rat kidney *in vivo* and in cultivated renal epithelial cells *in vitro*. Toxicology.

[77] Zepnik H., Volkel W., Dekant W. (2003). Toxicokinetics of the mycotoxin ochratoxin A in F 344 rats after oral administration. Toxicol. Appl. Pharmacol..

[78] Cavin C., Delatour T., Marin-Kuan M., Holzhauser D., Higgins L., Bezencon C., Guignard G., Junod S., Richoz-Payot J., Gremaud E. (2007). Reduction in antioxidant defenses may contribute to ochratoxin a toxicity and carcinogenicity. Toxicol. Sci..

[79] Bartsch H., Nair J. (2004). Oxidative stress and lipid peroxidation-derived DNA-lesions in inflammation driven carcinogenesis. Cancer Detect. Prev..

[80] Huang Y., Li W., Kong A.N. (2012). Anti-oxidative stress regulator NF-E2-related factor 2 mediates the adaptive induction of antioxidant and detoxifying enzymes by lipid peroxidation metabolite 4-hydroxynonenal. Cell Biosci..

[81] Boesch-Saadatmandi C., Wagner A.E., Graeser A.C., Hundhausen C., Wolffram S., Rimbach G. (2009). Ochratoxin A impairs Nrf2-dependent gene expression in porcine kidney tubulus cells. J. Anim. Physiol. Anim. Nutr..

[82] Pfohl-Leszkowicz A., Manderville R.A. (2007). Ochratoxin A: An overview on toxicity and carcinogenicity in animals and humans. Mol. Nutr. Food Res..

[83] Chaudhary M., Rao P.V. (2010). Brain oxidative stress after dermal and subcutaneous exposure of T-2 toxin in mice. Food Chem. Toxicol..

[84] Meissonnier G.M., Pinton P., Laffitte J., Cossalter A.M., Gong Y.Y., Wild C.P., Bertin G., Galtier P., Oswald I.P. (2008). Immunotoxicity of aflatoxin B1: Impairment of the cell-mediated response to vaccine antigen and modulation of cytokine expression. Toxicol. Appl. Pharmacol..

[85] Seeboth J., Solinhac R., Oswald I.P., Guzylack-Piriou L. (2012). The fungal T-2 toxin alters the activation of primary macrophages induced by TLR-agonists resulting in a decrease of the inflammatory response in the pig. Vet. Res..

[86] Mary V.S., Theumer M.G., Arias S.L., Rubinstein H.R. (2012). Reactive oxygen species sources and biomolecular oxidative damage induced by aflatoxin B1 and fumonisin B1 in rat spleen mononuclear cells. Toxicology.

[87] Theumer M.G., Canepa M.C., Lopez A.G., Mary V.S., Dambolena J.S., Rubinstein H.R. (2010). Subchronic mycotoxicoses in Wistar rats: Assessment of the *in vivo* and *in vitro* genotoxicity induced by fumonisins and aflatoxin b(1), and oxidative stress biomarkers status. Toxicology.

[88] Grenier B., Loureiro-Bracarense A.P., Lucioli J., Pacheco G.D., Cossalter A.M., Moll W.D., Schatzmayr G., Oswald I.P. (2011). Individual and combined effects of subclinical doses of deoxynivalenol and fumonisins in piglets. Mol. Nutr. Food Res..

[89] Schutze N., Lehmann I., Bonisch U., Simon J.C., Polte T. (2010). Exposure to mycotoxins increases the allergic immune response in a murine asthma model. Am. J. Respir. Crit. Care Med..

[90] Wichmann G., Herbarth O., Lehmann I. (2002). The mycotoxins citrinin, gliotoxin, and patulin affect interferon-gamma rather than interleukin-4 production in human blood cells. Environ. Toxicol..

[91] Luft P., Oostingh G.J., Gruijthuijsen Y., Horejs-Hoeck J., Lehmann I., Duschl A. (2008). Patulin influences the expression of Th1/Th2 cytokines by activated peripheral blood mononuclear cells and T cells through depletion of intracellular glutathione. Environ. Toxicol..

[92] Johannessen L.N., Nilsen A.M., Lovik M. (2007). Mycotoxin-induced depletion of intracellular glutathione and altered cytokine production in the human alveolar epithelial cell line A549. Toxicol. Lett..

[93] Peterson J.D., Herzenberg L.A., Vasquez K., Waltenbaugh C. (1998). Glutathione levels in antigen-presenting cells modulate Th1 *versus* Th2 response patterns. Proc. Natl. Acad. Sci. USA.

[94] Murata Y., Ohteki T., Koyasu S., Hamuro J. (2002). IFN-γand pro-inflammatory cytokine production by antigen-presenting cells is dictated by intracellular thiol redox status regulated by oxygen tension. Eur. J. Immunol..

[95] Fitzpatrick A.M., Teague W.G., Burwell L., Brown M.S., Brown L.A. (2011). Glutathione oxidation is associated with airway macrophage functional impairment in children with severe asthma. Pediatr. Res..

[96] Morris D., Guerra C., Khurasany M., Guilford F., Saviola B., Huang Y., Venketaraman V. (2013). Glutathione supplementation improves macrophage functions in hiv. J. Interferon Cytokine Res..

[97] Ballatori N., Krance S.M., Notenboom S., Shi S., Tieu K., Hammond C.L. (2009). Glutathione dysregulation and the etiology and progression of human diseases. Biol. Chem..

[98] Novi A.M., Florke R., Stukenkemper M. (1982). Glutathione and aflatoxin-B1-induced liver tumors: Requirement for an intact glutathione molecule for regression of malignancy in neoplastic tissue. Ann. N. Y. Acad. Sci..

[99] Hope J. (2013). A review of the mechanism of injury and treatment approaches for illness resulting from exposure to water-damaged buildings, mold, and mycotoxins. Sci. World J..

